# Evidence of Benefit of Telerehabitation After Orthopedic Surgery: A Systematic Review

**DOI:** 10.2196/jmir.6836

**Published:** 2017-04-28

**Authors:** Jose Manuel Pastora-Bernal, Rocio Martín-Valero, Francisco Javier Barón-López, María José Estebanez-Pérez

**Affiliations:** ^1^ Faculty of Health Sciences Physiotherapy University of Málaga Malaga Spain; ^2^ Faculty of Nursery and Physiotherapy Department of Nursery and Physiotherapy University of Cadiz Cadiz Spain; ^3^ Rehabilitación Costa del Sol Fisioterapia Private Centre Fuengirola Spain

**Keywords:** telerehabilitation, orthopedic surgery, musculoskeletal disorders, systematic review, telemedicine, mobile health, mHealth, telehealth, physiotherapy

## Abstract

**Background:**

In addition to traditional physiotherapy, studies based on telerehabilitation programs have published the results of effectiveness, validity, noninferiority, and important advantages in some neurological, cognitive, and musculoskeletal disorders, providing an opportunity to define new social policies and interventions.

**Objectives:**

The aim of this systematic review is to investigate the effects of telerehabilitation after surgical procedures on orthopedic conditions as well as to describe how interventions are designed and to determine whether telerehabilitation is comparable with conventional methods of delivery. This systematic review summarizes the levels of evidence and grades of recommendation regarding telerehabilitation intervention (synchronous or asynchronous provided via the telerehabilitation medium, either in conjunction with, or in isolation of, other treatment interventions) after surgical procedures on orthopedic conditions.

**Methods:**

Study quality was assessed using the Physiotherapy Evidence Database (PEDro) scores and grade of recommendation following the recommendation of the Oxford Centre for Evidence-Based Medicine.

**Results:**

We found 3 studies with PEDro scores between 6 and 8, which is considered as level 1 evidence (good; 20% [3/15]), 4 studies with a score of 5, which is considered as level 2 evidence (acceptable; 27% [4/15]), and the remaining 8 studies had scores of 4 or less, which is considered (poor; 53% [8/15]). A total of 1316 participants received telerehabilitation intervention in the selected studies, where knee and hip replacement were 75% of all the studies. Strong and moderate grades of evidence (grade of recommendation A–B) were found in knee and hip replacement interventions. Studies on the upper limb were 25% of the studies, but only 1 study presented a moderate grade of evidence (grade of recommendation B) and the rest were of poor methodological quality with weak evidence (grade of recommendation C).

**Conclusions:**

Conclusive evidence on the efficacy of telerehabilitation for treatment after an orthopedic surgery, regardless of pathology, was not obtained. We found strong evidence in favor of telerehabilitation in patients following total knee and hip arthroplasty and limited evidence in the upper limb interventions (moderate and weak evidence). Future research needs to be more extensive and conclusive. To the best of the authors’ knowledge, this is the first attempt at evaluating the quality of telerehabilitation intervention research after surgical procedures on orthopedic conditions in a systematic review. Clinical messages and future research recommendations are included in the review.

## Introduction

The increasing availability of low-cost Internet and communication technologies has boosted the opportunity to apply technology-based solutions to provide health services during hospitalization and after discharge from hospital [1]. With the general ageing of the population—at least in industrialized countries—and the limited resources devoted to public health, the development of new rehabilitation models and practices seems mandatory in order to cope with the change in population needs [2,3]. Telerehabilitation, one of the emerging fields of telemedicine, is defined as a set of tools, procedures, and protocols to deliver the rehabilitation process remotely [4]. It will be increasingly important to develop scalable and sustainable telerehabilitation programs [5].

The growing demand for rehabilitation can result in increased costs and longer waiting lists, becoming a threat to the sustainability of health care services [6]. Telerehabilitation can help with this issue by discharging patients from points of care while improving their adherence to treatment [6]. Recent telerehabilitation studies have addressed different approaches: feasibility and efficacy, patients’ and professionals’ satisfaction, and cost analysis studies. This includes neurological diseases [7-10], stroke patients [11-13], intensive care unit [14], breast cancer [15], COPD [16], and musculoskeletal disorders [17-22]. Although trials supporting the role of telerehabilitation continue to emerge, implementation has been slow [23].

The scientific community believes that telerehabilitation will play an important role in improving, or at least maintaining, the continuity of rehabilitation care and services as they are reorganized, as it is able to increase the efficiency of the services while containing costs [24].

Early systematic reviews have pointed out that, despite the growing number of telerehabilitation experiences worldwide, evidence of clinical and economic effectiveness is still lacking [2,25]. In addition, these reviews have commented on the lack of methodological rigor and variability of approaches used in telerehabilitation studies [4]. With the growing number of telerehabilitation programs and with emerging databases providing potentially useful and reliable data on clinical outcomes [26], it is reasonable to expect that the number of scientific publications will rapidly increase [27], along with the number of systematic reviews on the topic [4]. Systematic reviews that included telerehabilitation interventions in musculoskeletal conditions yielded a few eligible trials, so efficacy remains inconclusive [1,28]. Telerehabilitation shows promise in many fields, but compelling evidence of the benefit for, and impact on, routine rehabilitation programs is still limited. There is a need for detailed, better-quality studies and for studies on the use of telerehabilitation in routine care [26].

Musculoskeletal injuries are frequent events in routine care and are the most common source of chronic pain and disability [29]. Orthopedic surgeries are experiencing some of the greatest growth rates in developed nations across the world. A study from 2014 found that the most common inpatient operating room procedure in the United States involved the musculoskeletal system [30]. Therapeutic exercises are commonly prescribed following a surgery in an attempt to maximize functional outcome [31]. Physical therapists have been utilizing therapeutic exercises since the conception of the profession, and they have been demonstrated to be fundamental in improving function, performance, and disability [32]. Telerehabilitation offers the possibility to develop therapeutic exercise at a distance, among others.

Recently, a systematic review concluded that telerehabilitation is promising and highlighted the fact that for those individuals who are unable to attend traditional face-to-face services, particularly following elective orthopedic surgical procedures [33], telerehabilitation should be considered as a viable option in the holistic management of their musculoskeletal condition [33].

Despite the existence of systematic reviews on telerehabilitation interventions, none of them has explored post-surgical rehabilitation in musculoskeletal injuries. Therefore, the aim of this paper is to investigate the effects of telerehabilitation after surgical procedures on orthopedic conditions, as well as to describe how interventions are designed and to determine whether telerehabilitation is comparable with conventional methods of delivery within this population. We have considered all forms of interventions that use telecommunications technology to telerehabilitation interventions.

## Methods

### Search Strategy and Eligibility Criteria

This review has been carried out following the PRISMA 2009 guidelines [34]. The review protocol was registered with an international registration database [PROSPERO, Registration Number: CRD42016047846].

As with most of the recent systematic reviews on the topic [2,26,27,33,35], the following literature searches were performed to identify all possible studies that could help answer the research question. MEDLINE, Physiotherapy Evidence Database (PEDro), Scopus, PsycINFO, Web of Knowledge, CINAHL, SPORTDiscus, Directory of Open Access Journals (DOAJ), Cochrane, Embase, Academic Search Complete, Fuente Académica, and Consejo Superior de Investigaciones Científicas (CSIC) were searched. In addition, the search was performed in a relevant bibliographic database from the University of Málaga.

The initial search was carried out in June 2016, and was completed with a new search to update the review in October 2016. The following combinations of keywords were used: telerehabilitation, telerehabilitation physiotherapy, post-surgery telerehabilitation, musculoskeletal disorders, systematic review, telemedicine (mobile health or health, mobile or mHealth or telehealth or eHealth), telemedicine physiotherapy program. The limits of searches were studies published between 2000 and 2016.

**Figure 1:**
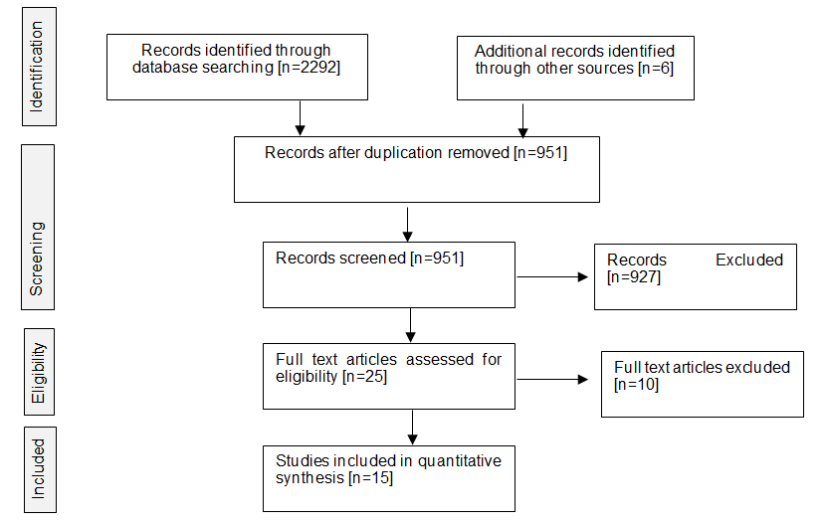
Flowchart.

The main steps related to the search phase are reported in Figure 1 using the PRISMA flow diagram [36]. After the application of the selected keywords, the entire set of records was analyzed to identify eventual duplication of articles retrieved from different sources; the remaining articles were then assessed in full text for eligibility so as to identify all those matching the inclusion criteria (see Multimedia Appendix 1).

Two authors (JMPB and RMV) independently screened the titles and abstracts of all records retrieved using the database search strategy. The full text was obtained if further information was required to determine eligibility, or if uncertainty prevailed between authors. For trials published in a language other than English or Spanish, a translated version of the abstract was sourced to determine eligibility.

Disagreements between authors were initially resolved via discussion, and then by consultation with a third reviewer (FJBL).

### Eligibility Criteria: Inclusion and Exclusion Criteria

Eligibility criteria were based on the PICOS framework [37], as follows:

#### Participants

Adults (≥18 years) with telerehabilitation services after surgical procedures as a result of a primary orthopedic condition. Trials in which the participant’s condition was secondary to a diagnosed health condition that was not primarily musculoskeletal in nature (eg, hand or shoulder dysfunction following stroke) were excluded.

#### Intervention

Any treatment intervention, synchronous or asynchronous, provided via a telerehabilitation medium, phone counseling, interactive virtual system, or gaming, either in conjunction with, or in isolation of, other treatment interventions was included.

#### Comparison

All trials were required to have a comparison group (of the same condition), where options included (but were not restricted to) face-to-face treatment or usual care. The comparison group could not be an alternative form of telerehabilitation. A pilot clinical trial without a comparison group was included if a telerehabilitation intervention had been carried out among participants.

#### Outcomes

Any clinical outcome, including measurements based on pain, quality of life, disability or function (physical, social, or psychological), was analyzed. Economic and cost-utility outcomes were not analyzed, nor were patient and clinician satisfaction or those outcomes measuring adherence to, or compliance with, rehabilitation programs.

### Study Design

All types of study designs were considered: randomized clinical trials (RCT), clinical trials (CT), case reports, controlled clinical trial, and pilot study. Articles that were limited to describing the feasibility and fit-out of telerehabilitation interventions were excluded.

For all eligible trials, data extraction was independently completed by 2 authors (JMPB and MJEP), and was cross-checked for consistency by a third author (RMV). The primary authors of eligible trials were contacted when information was considered to be missing for either the quality assessment or data extraction process.

### Evaluation of Methodological Quality, Level of Evidence, and Grade of Recommendation

An important step in conducting a systematic review is to assess the methodological quality of each included trial. In addition, reporting methodological quality provides clinicians with information about whether the results of clinical trials should influence their clinical practice. A valid way of assessing the methodological quality of clinical trials is therefore essential [38].

Two independent reviewers [JMPB and RMV] completed the checklist based on the PEDro scale. The methodological quality and risk of bias were evaluated using the PEDro scale [38,39] based on the Delphi list [40]. It is considered a useful tool for carrying out the assessment methodology in scientific research.

The PEDro scale scores 10 items: random allocation, concealed allocation, similarity at baseline, subject blinding, therapist blinding, assessor blinding, >85% follow-up for at least one key outcome, intention-to-treat analysis, between-group statistical comparison for at least one key outcome, and point and variability measures for at least one key outcome. Items are scored as either present (1) or absent (0) and a score out of 10 is obtained by summation [38]. The scale includes an additional item (eligibility criteria) to evaluate the external validity, but the score is not counted.

According to Moseley et al, studies with a PEDro score ≥5 will be considered at low risk of bias and high methodological quality [41].

A study with a PEDro score of ≥6 is considered to have level 1 evidence (6-8=good, 9-10=excellent) and a study with a score of ≤5 is considered to have level 2 evidence (4-5=acceptable, <4=poor) [42].

Levels of evidence help us target the search at the type of evidence that is most likely to provide a reliable answer. They have been designed so that they can be used as a shortcut for busy clinicians, researchers, or patients to find the likely best evidence [43]. Grades of recommendation describe the strength and therefore value of the evidence relative to how rigorous the study was (see Tables 1 and 2) [44].

**Table 1:** Based on evidence-based medicine working group [44].

Grades of recommendation		Strength of evidence
A	Strong Evidence	A preponderance of level I and/or level II studies support the recommendation. This must include at least 1 level I study.
B	Moderate Evidence	A single high-quality randomized controlled trial or a preponderance of level II studies support the recommendation
C	Weak Evidence	A single level II study or a preponderance of level III and IV studies including statements of consensus by content experts support the recommendation
D	Conflicting Evidence	Higher-quality studies conducted on this topic disagree with respect to their conclusions. The recommendation is based on these conflicting studies
E	Theoretical/Foundational Evidence	A preponderance of evidence from animal or cadaver studies, from conceptual models/principles, or from basic sciences/bench research support this conclusion
F	Expert Opinion	Best practice based on the clinical Experience of the guidelines development team

**Table 2:** Based on grades of recommendation and levels of evidence for therapy or prevention. Material adapted from the recommendations at the center for evidence-based medicine in oxford [43].

Level of evidence	Strength of evidence
1a	Systematic review of (homogeneous) randomized controlled trials
1b	Individual randomized controlled trials (with narrow CIs)
2a	Systematic review of (homogeneous) cohort studies of “exposed” and “unexposed” subject
2b	Individual cohort study / Low-quality randomized controlled trials
3a	Systematic review of (homogeneous) case-control studies
3b	Individual case-control studies
4	Case Series, low-quality cohort or case-control studies
5	Expert opinion based on non systematic reviews of results or mechanistic studies

## Results

The main findings of this review are presented in Table 3, an evaluation of the methodological quality of the 15 studies selected according to the PEDro scale. Characteristics of the included studies are listed in Multimedia Appendix 1 showing the grades of recommendation, regarding the effectiveness, results, and effect size in the different outcomes of telerehabilitation services. A subgroup analysis by population and intervention is presented in Tables 4 and 5.

Studies included in the review had PEDro scores of 2-8, as shown in Table 3. Studies were considered of high enough methodological quality if they had a score of at least 5. This was based on the fact that studies with a score closer to 4 did not use a triple-blind methodology (subject, evaluator, and treatment provider) [45].

We found 3 studies [20,46,47] with PEDro scores between 6 and 8, which is considered level 1 evidence (good; 20% [3/15]), 4 studies [48-50,51] with a score of 5, which is considered level 2 evidence (acceptable; 27% [4/15]), and the remaining 8 studies [52-56,21,57,58] had scores of 4 or less, which is considered (poor; 53% [8/15]).

A total of 1316 participants received telerehabilitation intervention. Strong and moderate grades of evidence (grade of recommendation A-B) were found in knee and hip replacement interventions (80% of all the studies). Studies in the upper limb were 20% of the studies included but only 1 study presented a moderate grade of evidence (grade or recommendation B) and the rest were of poor methodological quality with weak evidence (grade of recommendation C).

The subgroup analysis by population shows us 8 articles focused on a total knee replacement population (50% of articles), 4 on total hip replacement (25% of articles), 1 on shoulder joint replacement (6% of articles), 1 on proximal humerus fractures (6% of articles), 1 on carpal tunnel release surgery (6% of articles), and 1 on rotator cuff tear (6% of articles). Another 3 telerehabilitation publications in the upper limbs (hand transplantation [case study]) [59], and hand surgery (preclinical trials and descriptive study) [60,61], have been identified in addition, but as no intervention on the subject has been published, they do not match the inclusion criteria for this review (See Multimedia Appendix 2).

In the subgroup analysis by telerehabilitation intervention (Table 5), we found strong evidence regardless of the intervention (videoconferencing, asynchronous, phone counseling, interactive virtual system and gaming, telerehabilitation system). Therefore, we may interpret that evidence level and degree of recommendation are related to studies design, and are not related to intervention.

We found great heterogeneity among the included studies. Sample size ranged between 5 [56] and 237 [53]. Telerehabilitation interventions included videoconferencing sessions [20,21,46,50,55], phone counseling [49,53], video games [62,63], asynchronous exercise videos, and interactive virtual systems [19,47,52,54-57].

Compared interventions also range between clinical protocol face-to-face physiotherapy [19,46,50,52,55], home physiotherapy visits and usual care at home [20,53,54], and a physiotherapy session followed by gaming [63].

Intervention duration ranged from 2 weeks [19] to 26 weeks [53] with follow-up periods from 13 sessions [56] to 9 months [49].

We found some homogeneous aspects in clinical outcomes primarily in the areas of function, quality of life, and specific daily life activities [20,46,54,63], and less assessment of disability (passive and active ranges of motion, balance, and muscle strength) and pain.

**Table 3:** Evaluation of methodological quality of the 15 selected studies.

PEDro scale criteria	Moffet et al [20]	Russell et al [46]	Bini et al [47]	Piqueras et al [51]	Tousignant et al [50]	Hørdam et al [49]	Fung et al [48]	Li et al [53]	Russell et al [54]	Eriksson et al [55]	Eisermann et al [52]	Antón et al [58]	Tousignant et al [64]	Heuser et al [56]	Macias et al [57]
Eligibility criteria^a^	Y^b^	Y	Y	Y	Y	Y	Y	Y	N^c^	Y	Y	Y	Y	Y	Y
Randomization	Y	Y	Y	Y	Y	Y	Y	N	Y	N	Y	N	N	N	N
Allocation concealed	Y	Y	Y	N	Y	Y	N	N	N	N	N	N	N	N	N
Baseline comparability	Y	Y	Y	Y	Y	Y	Y	Y	N	Y	N	N	N	N	N
Subject blinding	N	N	N	N	N	N	N	N	N	N	N	N	N	N	N
Therapist blinding	N	N	N	N	N	N	N	N	N	N	N	N	N	N	N
Evaluator blinding	Y	Y	N	Y	N	N	Y	N	Y	N	N	N	N	N	N
Appropriate continuation	Y	Y	Y	N	N	Y	Y	Y	Y	Y	N	Y	Y	Y	Y
Intention to treat	Y	Y	Y	N	N	N	N	N	N	Y	N	N	N	N	N
Comparison between groups	Y	Y	Y	Y	Y	Y	Y	Y	Y	Y	Y	N	N	N	N
Specific measurements and variability	Y	Y	Y	Y	Y	N	N	Y	N	N	Y	Y	Y	Y	Y
Total PEDro Score	8	8	7	5	5	5	5	4	4	4	3	2	2	2	2

^a^The eligibility criteria do not contribute to the total score.

^b^Y is Yes.

^c^N is No.

**Table 4:** Subgroup analysis by population.

Population	Authors and reference	Number of articles	Participants (n)	Articles with grade of recommendation A	Articles with grade of recommendation B	Articles with grade of recommendation C and D
				Level of evidence 1 (% of total articles)	Level of evidence 2 or 3 (% of total articles)	Level of evidence >3 (% of total articles)
Total knee arthroplasty	Moffet et al 2015 [20], Russell et al 2011 [46], Bini et al 2016 [47], Piqueras et al 2013 [19], Tousignant et al 2011 [50], Eisermann et al 2004 [52], Fung et al 2012 [48], Russell et al 2003 [54]	8	718	3 (19)	5 (31)	0
Total Hip Replacement	Hørdam et al 2009 [49], Eisermann et al 2004 [52], Li et al 2014 [53], Antón et al 2016 [58]	4	543	3 (19)	0	1 (6.25)
Shoulder joint replacement	Eriksson et al 2009 [55]	1	22	0	1 (6.25)	0
Proximal humerus fractures	Tousignant et al 2015 [64]	1	17	0	0	1 (6.25)
Carpal tunnel release surgery	Heuser et al 2007 [56]	1	5	0	0	1 (6.25)
Rotator Cuff Tear	Macías-Hernández et al 2016 [57]	1	11	0	0	1 (6.25)
Total %		16 (Eisermann et al included knee and hip population)	1316	38.00	37.25	25

**Table 5:** Subgroup analysis by intervention.

Intervention	Authors and reference	Number of articles	Participants (n)	Articles with level of evidence 1 and grade of recommendation A (% of total articles)	Articles with level of evidence 2 or 3 and grade of recommendation B (% of total articles)	Articles with level of evidence >3 and grade of recommendation C and D (% of total articles)
Videoconferencing (real-time)	Moffet et al 2015 [20], Russell et al 2011 [46], Tousignant et al 2011 [50], Eriksson et al 2009 [55], Tousignant et al 2015 [64]	5	357	3 (21.4)	1 (6.6)	1 (6.6)
Asynchronous videos program	Bini et al 2016 [47], Eisermann et al 2004 [52], Macías-Hernández et al 2016 [57]	3	336	2 (14.3)	0	1 (6.6)
Education sessions by telephone	Hørdam et al 2009 [49], Li et al 2014 [53]	2	398	2 (14.3)	0	0
Interactive virtual TR system & gaming	Piqueras et al 2013 [19], Fung et al 2012 [48], Russell et al 2003 [54], Antón et al 2016 [58], Heuser et al 2007 [56]	5	225	2 (14.3)	1 (6.6)	2 (13.3)
Total %		15	1316	60	13.33	26.67

## Discussion

### Principal Findings

This review confirms the strong evidence in favor of telerehabilitation among patients undergoing total knee and hip arthroplasty and the limited evidence in the upper limb (moderate and weak evidence).

To the best of our knowledge, this is the first review focused on telerehabilitation research after surgical procedures on orthopedic conditions. This systematic review applied a qualitative evaluation to provide a wider picture of currently available evidence.

First, we will discuss the contributions of the first systematic reviews on the topic. Second, we discuss the generalizations, previous results, and future recommendations. Third, we discuss if results are extrapolated to the upper limb (the results of this review show poor-quality methodology and moderate and weak evidence). Finally, we discuss about the inherent difficulties in conducting telerehabilitation research and future research recommendations.

Regarding the first aspect noted in the discussion, the first systematic reviews, contributions concluded that better-quality studies are needed as well as studies on the use of telerehabilitation in routine care. Telerehabilitation research is generally not very good and there are many reviews that criticize this [2,26,27,33,35]. In our review, 60% of the included studies are of poor methodological quality with weak evidence.

Regarding the second aspect noted in the discussion, the most recent systematic reviews provide statements on the effects of telerehabilitation interventions. Two recent studies provide statements such as, “there is a strong positive effect for patients following orthopedic surgery” [1] and “there is unequivocal evidence that the management of musculoskeletal conditions via real-time telerehabilitation is effective in improving physical function, disability, and pain” [33]. We agree with these statements, but only and exclusively for some pathologies. Our systematic review shows that there is still insufficient evidence on upper limb surgeries’ telerehabilitation interventions.

Regarding the third aspect noted in the discussion: does this statement transfer to upper limb such as shoulder arthroscopy, carpal tunnel release surgery, hand surgery, or shoulder arthroplasty? Could this be extrapolated to the rehabilitation process in fractures or surgery interventions in upper and lower limbs? These unresolved clinical questions reaffirm the need to identify the available evidence in post-surgical rehabilitation with telerehabilitation interventions.

For this systematic review, we seek to find evidence of post-surgical telerehabilitation programs, with special emphasis on programs that can be integrated into clinical practice.

In our review, telerehabilitation research in the upper limb (shoulder joint replacement, proximal humerus fractures, carpal tunnel release, and cuff rotator tears) presents moderate and weak levels of evidence. Notable is the judgment that none of the telerehabilitation studies in the upper limb included in this review present a high level of evidence and recommendation. There is still a very small database for telerehabilitation studies after a musculoskeletal surgery that provides useful data on clinical outcomes, especially in conditions other than the replacement of joints in the lower limb. Therefore, conclusive evidence on the efficacy of telerehabilitation for treatment after an orthopedic surgery, regardless of pathology, was not obtained.

Research background has been used to discuss the strengths of telerehabilitation and the opportunities for future interventions and policies. Regarding the final aspect noted in the discussion, what are the inherent difficulties in conducting telerehabilitation research?

During the search, we observed a number of studies that provide descriptions of telerehabilitation interventions of low methodological quality. No validated clinical outcomes, too small a sample size, and a lack of comparison group are frequently found. Moreover, differences in telerehabilitation interventions, treatment period, and follow-up, create doubts in identifying whether the telerehabilitation gives comparable or better results.

A frequent problem in studies of telerehabilitation is the lack of blinding of therapists and patients. There is evidence that in clinical trials where allocation is not concealed and assessors, therapists, and participants are not blinded, a larger effect of intervention is reported than in higher quality trials with adequate blinding procedures [65].

It may be that there are good-quality studies the publication of which has been delayed; however, our findings are aligned and consistent with the most recent revisions regarding the need for future research needs to have stronger and more solid studies.

One of the biases identified is that telerehabilitation groups have more frequent contact with health professionals and with the intervention (especially in videoconferencing and phone contact), so they are likely to receive additional services. This creates biases whether the positive results are related to a more elaborate program than really with the interventions method.

How could this be addressed in future research? As blinding of patients and therapists is not possible in telerehabilitation interventions, several methodological aspects are fundamental for future research.

Telerehabilitation interventions should be conceptualized, coded, classified, and grouped in a similar way to physiotherapy technique codes, enabling identification in detail when the effect is due to the type of intervention. Comparison group must be the actual best evidence treatment for the same condition that allows identification of whether telerehabilitation offers better or comparable outcomes. Telerehabilitation frequency must be the same as the control group to avoid biases related to a more elaborate program. Greater homogeneous is needed especially in terms of type, duration, and intervention follow-up for each specific pathologies. Studies that show negative results should be published, avoiding publication biases. Large sample size and improvement in study quality (allocated and evaluator blinding) must be addressed. Orthopedic conditions and musculoskeletal injuries different to replacement joints in lower limbs need quality research.

### Clinical Messages

High-methodological-quality studies should be conducted to confirm that telerehabilitation shows clinically relevant outcomes after surgery in orthopedic and musculoskeletal injuries, especially in upper limbs. Telerehabilitation appears to be an effective alternative to face-to-face service delivery after hospital discharge of patients following total knee arthroplasty and hip replacement. Clinical outcomes are comparable and not inferior. Despite some limitations, there seem to be clear benefits from physiotherapy at a distance regardless with the telerehabilitation technique it offers (videoconferencing, phone intervention, asynchronous video exercise programs, or gaming). Future challenges include identifying whether positive results are due to the type of intervention or the increased frequency and intensity that telerehabilitation allow.

Future research recommendations for telerehabilitation should include high-quality studies with clear conclusions and statements that could improve health interventions and health policies.

## Appendix

Multimedia Appendix 1 Characteristics of the included studies.

Multimedia Appendix 2 Excluded articles.

## Abbreviations

CT: clinical trials
RCT: randomized controlled trial

## References

[1] Agostini M, Moja L, Banzi R, Pistotti V, Tonin P, Venneri A, Turolla A (2015). Telerehabilitation and recovery of motor function: a systematic review and meta-analysis. J Telemed Telecare.

[2] Rogante M, Grigioni M, Cordella DG, Giacomozzi Claudia (2010). Ten years of telerehabilitation: a literature overview of technologies and clinical applications. NeuroRehabilitation.

[3] Zampolini M, Todeschini E, Bernabeu GM, Hermens H, Ilsbroukx S, Macellari V, Magni R, Rogante M, Scattareggia MS, Vollenbroek M, Giacomozzi C (2008). Tele-rehabilitation: present and future. Ann Ist Super Sanita.

[4] Rogante M, Kairy D, Giacomozzi C, Grigioni M (2015). A quality assessment of systematic reviews on telerehabilitation: what does the evidence tell us?. Ann Ist Super Sanita.

[5] Dinesen B, Nonnecke B, Lindeman D, Toft E, Kidholm K, Jethwani K, Young HM, Spindler H, Oestergaard CU, Southard JA, Gutierrez M, Anderson N, Albert NM, Han JJ, Nesbitt T (2016). Personalized telehealth in the future: a global research agenda. J Med Internet Res.

[6] Ruiz-Fernandez D, Marín-Alonso O, Soriano-Paya A, García-Pérez JD (2014). eFisioTrack: a telerehabilitation environment based on motion recognition using accelerometry. ScientificWorldJournal.

[7] Houlihan BV, Jette A, Friedman RH, Paasche-Orlow M, Ni P, Wierbicky J, Williams K, Ducharme S, Zazula J, Cuevas P, Rosenblum D, Williams S (2013). A pilot study of a telehealth intervention for persons with spinal cord dysfunction. Spinal Cord.

[8] Jelcic N, Agostini M, Meneghello F, Bussè C, Parise S, Galano A, Tonin P, Dam M, Cagnin A (2014). Feasibility and efficacy of cognitive telerehabilitation in early Alzheimer's disease: a pilot study. Clin Interv Aging.

[9] Paul L, Coulter EH, Miller L, McFadyen A, Dorfman J, Mattison PG (2014). Web-based physiotherapy for people moderately affected with Multiple Sclerosis; quantitative and qualitative data from a randomized, controlled pilot study. Clin Rehabil.

[10] Hoffmann T, Russell T, Thompson L, Vincent A, Nelson M (2008). Using the internet to assess activities of daily living and hand function in people with Parkinson's disease. NeuroRehabilitation.

[11] Lin K, Chen C, Chen Y, Huang W, Lai J, Yu S, Chang Y (2014). Bidirectional and multi-user telerehabilitation system: clinical effect on balance, functional activity, and satisfaction in patients with chronic stroke living in long-term care facilities. Sensors (Basel).

[12] Langan J, Delave K, Phillips L, Pangilinan P, Brown SH (2013). Home-based telerehabilitation shows improved upper limb function in adults with chronic stroke: a pilot study. J Rehabil Med.

[13] Deng H, Durfee WK, Nuckley DJ, Rheude BS, Severson AE, Skluzacek KM, Spindler KK, Davey CS, Carey JR (2012). Complex versus simple ankle movement training in stroke using telerehabilitation: a randomized controlled trial. Phys Ther.

[14] Jackson JC, Ely EW, Morey MC, Anderson VM, Denne LB, Clune J, Siebert CS, Archer KR, Torres R, Janz D, Schiro E, Jones J, Shintani AK, Levine B, Pun BT, Thompson J, Brummel NE, Hoenig H (2012). Cognitive and physical rehabilitation of intensive care unit survivors: results of the RETURN randomized controlled pilot investigation. Crit Care Med.

[15] Galiano-Castillo N, Ariza-García A, Cantarero-Villanueva I, Fernández-Lao C, Díaz-Rodríguez L, Legerén-Alvarez M, Sánchez-Salado C, Del-Moral-Avila R, Arroyo-Morales M (2013). Telehealth system (e-CUIDATE) to improve quality of life in breast cancer survivors: rationale and study protocol for a randomized clinical trial. Trials.

[16] Bedra M, McNabney M, Stiassny D, Nicholas J, Finkelstein J (2013). Defining patient-centered characteristics of a telerehabilitation system for patients with COPD. Stud Health Technol Inform.

[17] Bennell KL, Rini C, Keefe F, French S, Nelligan R, Kasza J, Forbes A, Dobson F, Abbott JH, Dalwood A, Vicenzino B, Harris A, Hinman RS (2015). Effects of adding an internet-based pain coping skills training protocol to a standardized education and exercise program for people with persistent hip pain (HOPE trial): randomized controlled trial protocol. Phys Ther.

[18] Truter P, Russell T, Fary R (2014). The validity of physical therapy assessment of low back pain via telerehabilitation in a clinical setting. Telemed J E Health.

[19] Piqueras M, Marco E, Coll M, Escalada F, Ballester A, Cinca C, Belmonte R, Muniesa JM (2013). Effectiveness of an interactive virtual telerehabilitation system in patients after total knee arthoplasty: a randomized controlled trial. J Rehabil Med.

[20] Moffet H, Tousignant M, Nadeau S, Mérette C, Boissy P, Corriveau H, Marquis F, Cabana F, Ranger P, Belzile ÉL, Dimentberg R (2015). In-Home telerehabilitation compared with face-to-face rehabilitation after total knee arthroplasty: a noninferiority randomized controlled trial. J Bone Joint Surg Am.

[21] Tousignant M, Giguère A-M, Morin M, Pelletier J, Sheehy A, Cabana F (2014). In-home telerehabilitation for proximal humerus fractures: a pilot study. Int J Telerehabil.

[22] Bedra M, Finkelstein J (2015). Feasibility of post-acute hip fracture telerehabilitation in older adults. Stud Health Technol Inform.

[23] Shulver W, Killington M, Morris C, Crotty M (2017). 'Well, if the kids can do it, I can do it': older rehabilitation patients' experiences of telerehabilitation. Health Expect.

[24] Theodoros D, Russell T (2008). Telerehabilitation: current perspectives. Stud Health Technol Inform.

[25] Kairy D, Lehoux P, Vincent C, Visintin M (2009). A systematic review of clinical outcomes, clinical process, healthcare utilization and costs associated with telerehabilitation. Disabil Rehabil.

[26] Hailey D, Roine R, Ohinmaa A, Dennett L (2011). Evidence of benefit from telerehabilitation in routine care: a systematic review. J Telemed Telecare.

[27] Laver KE, Schoene D, Crotty M, George S, Lannin NA, Sherrington C (2013). Telerehabilitation services for stroke. Cochrane Database Syst Rev.

[28] Pietrzak E, Cotea C, Pullman S, Nasveld P (2013). Self-management and rehabilitation in osteoarthritis: is there a place for internet-based interventions?. Telemed J E Health.

[29] Russell TG, Blumke R, Richardson B, Truter P (2010). Telerehabilitation mediated physiotherapy assessment of ankle disorders. Physiother Res Int.

[30] Fingar K, Stocks C, Weiss A, Steiner C (2014). HCUP Statistical Brief # 186 Internet.

[31] Eriksson L, Lindström B, Ekenberg L (2011). Patients' experiences of telerehabilitation at home after shoulder joint replacement. J Telemed Telecare.

[32] Saunders DG (2007). Therapeutic exercise. Clin Tech Small Anim Pract.

[33] Cottrell MA, Galea OA, O'Leary SP, Hill AJ, Russell TG (2016). Real-time telerehabilitation for the treatment of musculoskeletal conditions is effective and comparable to standard practice: A systematic review and meta-analysis. Clin Rehabil.

[34] Liberati A, Altman DG, Tetzlaff J, Mulrow C, Gøtzsche PC, Ioannidis JP, Clarke M, Devereaux PJ, Kleijnen J, Moher D (2009). The PRISMA statement for reporting systematic reviews and meta-analyses of studies that evaluate healthcare interventions: explanation and elaboration. BMJ.

[35] Hailey D, Roine R, Ohinmaa A, Dennett L (2013). The status of telerehabilitation in neurological applications. J Telemed Telecare.

[36] Shamseer L, Moher D, Clarke M, Ghersi D, Liberati A, Petticrew M, Shekelle P, Stewart LA, PRISMA-P Group (2015). Preferred reporting items for systematic review and meta-analysis protocols (PRISMA-P) 2015: elaboration and explanation. BMJ.

[37] (2009). Systematic Reviews: CRD's Guidance for Undertaking Reviews in Healthcare.

[38] de Morton Na (2009). The PEDro scale is a valid measure of the methodological quality of clinical trials: a demographic study. Aust J Physiother.

[39] Maher CG, Sherrington C, Herbert RD, Moseley AM, Elkins M (2003). Reliability of the PEDro scale for rating quality of randomized controlled trials. Phys Ther.

[40] Verhagen A, de Vet HC, de Bie RA, Kessels A, Boers M, Bouter L, Knipschild P G (1998). The Delphi list: a criteria list for quality assessment of randomized clinical trials for conducting systematic reviews developed by Delphi consensus. J Clin Epidemiol.

[41] Moseley A, Herbert R, Sherrington C, Maher C (2002). Evidence for physiotherapy practice: a survey of the Physiotherapy Evidence Database (PEDro). Aust J Physiother.

[42] Foley NC, Teasell RW, Bhogal SK, Speechley MR (2003). Stroke rehabilitation evidence-based review: methodology. Top Stroke Rehabil.

[43] (2011). CEBM.

[44] Guyatt GH, Sackett DL, Sinclair JC, Hayward R, Cook DJ, Cook RJ (1995). Users' guides to the medical literature. IX. A method for grading health care recommendations. Evidence-Based Medicine Working Group. JAMA.

[45] Martin-Valero R, De La Casa AM, Casuso-Holgado M, Heredia-Madrazo A (2015). Systematic review of inspiratory muscle training after cerebrovascular accident. Respir Care.

[46] Russell T, Buttrum P, Wootton R, Jull G (2011). Internet-based outpatient telerehabilitation for patients following total knee arthroplasty: a randomized controlled trial. J Bone Joint Surg Am.

[47] Bini SA, Mahajan J (2017). Clinical outcomes of remote asynchronous telerehabilitation are equivalent to traditional therapy following total knee arthroplasty: A randomized control study. J Telemed Telecare.

[48] Fung V, Ho A, Shaffer J, Chung E, Gomez M (2012). Use of Nintendo Wii Fit™ in the rehabilitation of outpatients following total knee replacement: a preliminary randomised controlled trial. Physiotherapy.

[49] Hørdam B, Sabroe S, Pedersen PU, Mejdahl S, Søballe K (2010). Nursing intervention by telephone interviews of patients aged over 65 years after total hip replacement improves health status: a randomised clinical trial. Scand J Caring Sci.

[50] Tousignant M, Moffet H, Boissy P, Corriveau H, Cabana F, Marquis F (2011). A randomized controlled trial of home telerehabilitation for post-knee arthroplasty. J Telemed Telecare.

[51] Piqueras M, Marco E, Coll M, Escalada F, Ballester A, Cinca C, Belmonte R, Muniesa JM (2013). Effectiveness of an interactive virtual telerehabilitation system in patients after total knee arthoplasty: a randomized controlled trial. J Rehabil Med.

[52] Eisermann U, Haase I, Kladny B (2004). Computer-aided multimedia training in orthopedic rehabilitation. Am J Phys Med Rehabil.

[53] Li L, Gan Y, Zhang L, Wang Y, Zhang F, Qi J (2014). The effect of post-discharge telephone intervention on rehabilitation following total hip replacement surgery. Int J Nurs Sci.

[54] Russell TG, Buttrum P, Wootton R, Jull GA (2003). Low-bandwidth telerehabilitation for patients who have undergone total knee replacement: preliminary results. J Telemed Telecare.

[55] Eriksson L, Lindström B, Gard G, Lysholm J (2009). Physiotherapy at a distance: a controlled study of rehabilitation at home after a shoulder joint operation. J Telemed Telecare.

[56] Heuser A, Kourtev H, Winter S, Fensterheim D, Burdea G, Hentz V, Forducey P (2007). Telerehabilitation using the Rutgers Master II glove following carpal tunnel release surgery: proof-of-concept. IEEE Trans Neural Syst Rehabil Eng.

[57] Macías-Hernández SI, Vásquez-Sotelo DS, Ferruzca-Navarro MV, Badillo SS, Gutiérrez-Martínez J, Núñez-Gaona MA, Meneses HA, Velez-Gutiérrez OB, Tapia-Ferrusco I, Soria-Bastida M, Coronado-Zarco R, Morones-Alba JD (2016). Proposal and evaluation of a telerehabilitation platform designed for patients with partial rotator cuff tears: a preliminary study. Ann Rehabil Med.

[58] Antón D, Nelson M, Russell T, Goñi A, Illarramendi A (2016). Validation of a Kinect-based telerehabilitation system with total hip replacement patients. J Telemed Telecare.

[59] Giansanti D, Morelli S, Maccioni G, Lanzetta M, Macellari V (2008). Health technology assessment of a homecare device for telemonitoring and telerehabilitation for patients after hand transplantation. Telemed J E Health.

[60] Placidi G (2007). A smart virtual glove for the hand telerehabilitation. Comput Biol Med.

[61] Burdea G, Popescu V, Hentz V, Colbert K (2000). Virtual reality-based orthopedic telerehabilitation. IEEE Trans Rehabil Eng.

[62] Antón D, Nelson M, Russell T, Goñi A, Illarramendi A (2016). Validation of a Kinect-based telerehabilitation system with total hip replacement patients. J Telemed Telecare.

[63] Antón D, Nelson M, Russell T, Goñi A, Illarramendi A (2016). Validation of a Kinect-based telerehabilitation system with total hip replacement patients. J Telemed Telecare.

[64] Tousignant M, Giguère A, Morin M, Pelletier J, Sheehy A, Cabana F (2014). In-home telerehabilitation for proximal humerus fractures: a pilot study. Int J Telerehabil.

[65] Egger M, Juni P, Bartlett C, Holenstein F, Sterne J (2003). How important are comprehensive literature searches and the assessment of trial quality in systematic reviews? Empirical study. Health Technol Assess.

[66] Levy CE, Silverman E, Jia H, Geiss M, Omura D (2015). Effects of physical therapy delivery via home video telerehabilitation on functional and health-related quality of life outcomes. J Rehabil Res Dev.

[67] Spangehl M (2015). Is it time for telerehabilitation to go mainstream?. J Bone Joint Surg Am.

[68] Peterson C, Watzlaf V (2014). Telerehabilitation store and forward applications: a review of applications and privacy considerations in physical and occupational therapy practice. Int J Telerehabil.

[69] Sharareh B, Schwarzkopf R (2014). Effectiveness of telemedical applications in postoperative follow-up after total joint arthroplasty. J Arthroplasty.

[70] Bendixen RM, Levy C, Lutz BJ, Horn KR, Chronister K, Mann WC (2008). A telerehabilitation model for victims of polytrauma. Rehabil Nurs.

[71] Burdea G, Popescu V, Hentz V, Colbert K (2000). Virtual reality-based orthopedic telerehabilitation. IEEE Trans Rehabil Eng.

[72] Vuorenmaa M, Ylinen J, Piitulainen K, Salo P, Kautiainen H, Pesola M, Häkkinen A (2014). Efficacy of a 12-month, monitored home exercise programme compared with normal care commencing 2 months after total knee arthroplasty: a randomized controlled trial. J Rehabil Med.

[73] Girone M, Burdea G, Bouzit M, Popescu V, Deutsch JE (2000). Orthopedic rehabilitation using the “Rutgers ankle” interface. Stud Health Technol Inform.

