# Supplementary material for: Evidence of Benefit of Telerehabitation After Orthopedic Surgery: A Systematic Review
Source: J Med Internet Res. 2017 Apr 28;19(4):e142. doi: 10.2196/jmir.6836 (PMC5429438; doi:10.2196/jmir.6836)
Supplement: Multimedia Appendix 1 [file jmir_v19i4e142_app1.pdf]

Author (Year)	Population	Participants, n; (PEDro, type of evidence)	Recommendation Grade	Intervention	Outcome measures	TR[a] duration (weeks)	Follow-up (months)	Results
Moffet et al. 2015 [19]	Total knee arthroplasty (TKA)	205 (TR 104 / Con[b] 101) 8/10, RCT[c]	A	TR = Protocol-based physiotherapy via TR (videoconferencing) + HEP[d]<br>Con = protocol-based physiotherapy visit at home + HEP	(WOMAC[e])	8	4	Demonstrates the non-inferiority of in-home TR and supports its use as an effective alternative to face-to-face service delivery after hospital discharge of patients following a total knee arthroplasty (for 182 patients in per-protocol analysis): 21.6% (95% CI[n] –5.6%, 2.3%) for the total score–1.6% (95% CI –5.9%, 2.8%) for pain, –0.7% (95% CI –6.8%, 5.4%) for stiffness, and –1.8% (95% CI –5.9%, 2.3%) for function
Russell et al 2011 [46]	Total knee arthroplasty	65 (TR 31 / Con 34), 8/10, RCT	A	TR=clinical pathway protocol via TR videoconferencing, plus HEP<br>Con=clinical protocol face-to-face physiotherapy + HEP	WOMAC	6	1.5	Outcomes via TR at 6 weeks following a total knee arthroplasty were comparable with those after conventional rehabilitation. All participants had significant improvement in all outcome measures with the intervention (*P*<.01 for all). Better outcomes for the Patient-Specific Functional Scale and the stiffness subscale of the WOMAC were found in the TR group (*P*<.05)

[a]TR: telerehabilitation group.

[b]Con: control group.

[c]RCT: randomized clinical trial.

[d]HEP: home exercise program.

[e]WOMAC: Western Ontario & McMaster Universities Arthritis Scale.

[f]VAS: Visual Analogue Scale.

[g]KOOS: Knee injury and Osteoarthritis Outcome Score.

[h]PS: physical Function Short Form

[i]MCSI: minimal clinically significant improvement.

[j]PCS: physical component scores

[k]MCS: mental component scores

[l]ROM: range of motion.

[m]PRO: patient-reported outcome.

[n]CI: confidence interval.

[o]FIM: functional independence measures.

[p]SF-36: questionnaire short-form 36

[q]PF: physical function

[r]RF: role physical

[s]BP: bodily pain

[t]SF: social Function

[u]VT: vitality

[v]MH: mental Health

[w]FFbH: the Hanover Functional Ability Questionnaire

[x]StS: staffelstein Score

[y]HSS: hospital for special surgery score
