# Supplementary material for: Evidence of Benefit of Telerehabitation After Orthopedic Surgery: A Systematic Review
Source: J Med Internet Res. 2017 Apr 28;19(4):e142. doi: 10.2196/jmir.6836 (PMC5429438; doi:10.2196/jmir.6836)
Supplement: Multimedia Appendix 2 [file jmir_v19i4e142_app2.pdf]

Effects of physical therapy delivery via home video telerehabilitation on functional and health-related quality of life outcomes [62]	Population does not receive surgery. Doesn´t follow all inclusion criteria.
Is It Time for Telerehabilitation to Go Mainstream? [63]	Critical commentary on a TR article. Doesn´t follow all inclusion criteria.
Telerehabilitation store and forward applications: a review of applications and privacy considerations in physical and occupational therapy practice [64]	Descriptive article. Doesn´t follow all inclusion criteria
Effectiveness of telemedical applications in postoperative follow-up after total joint arthroplasty [65]	Satisfaction measures and medical visits. No clinical outcomes. Doesn´t follow all inclusion criteria.
A smart virtual glove for the hand telerehabilitation [60]	Descriptive article. Doesn´t follow all inclusion criteria.
A telerehabilitation model for victims of polytrauma [70]	Population does not receive surgery. Doesn´t follow all inclusion criteria.
Health technology assessment of a homecare device for telemonitoring and telerehabilitation for patients after hand transplantation [59]	Descriptive article. Doesn´t follow all inclusion criteria.
Virtual reality-based orthopedic telerehabilitation [71]	Descriptive article. Doesn´t follow all inclusion criteria.
Efficacy of a 12-month, monitored home exercise programme compared with normal care commencing 2 months after total knee arthroplasty: a randomized controlled trial [72]	No telerehabilitation intervention.
Orthopedic rehabilitation using the "Rutgers ankle" interface [73]	Population does not receive surgery. Doesn´t follow all inclusion criteria.

REFERENCES:

62. Levy CE, Silverman E, Jia H, Geiss M, Omura D. Effects of physical therapy delivery via home video telerehabilitation on functional and health-related quality of life outcomes. J Rehabil Res Dev [Internet]. 2015 [cited 2016 Jul 18];52[3]:361–70. Available from: http://www.ncbi.nlm.nih.gov/pubmed/26230650

63. Spangehl MJ, Ramos N, Wang E, Karia R, Hutzler L, Lajam C, et al. Is It Time for Telerehabilitation to Go Mainstream? J Bone Jt Surg Am. The American Orthopedic Association; 2015;97[14]:1717–22.

64. Peterson C, Watzlaf V. Telerehabilitation store and forward applications: a review of applications and privacy considerations in physical and occupational therapy practice. Int J telerehabilitation [Internet]. 2014 [cited 2016 Jul 18];6[2]:75–84. Available from: http://www.ncbi.nlm.nih.gov/pubmed/25945231

65. Sharareh B, Schwarzkopf R. Effectiveness of Telemedical Applications in Postoperative Follow-Up After Total Joint Arthroplasty. J Arthroplasty. 2014;29[5]:918–922.e1.

66. Bendixen RM, Levy C, Lutz BJ, Horn KR, Chronister K, Mann WC. A telerehabilitation model for victims of polytrauma. Rehabil Nurs [Internet]. [cited 2016 Jul 18];33[5]:215–20. Available from: http://www.ncbi.nlm.nih.gov/pubmed/18767403

67. Vuorenmaa M, Ylinen J, Piitulainen K, Salo P, Kautiainen H, Pesola M, et al. Efficacy of a 12-month, monitored home exercise programme compared with normal care commencing 2 months after total knee arthroplasty: A randomized controlled trial. J Rehabil Med [Internet]. 2013 Feb [cited 2016 Jul 18];46[January 2008]:166–72. Available from: http://www.ncbi.nlm.nih.gov/pubmed/24241606

68. Girone M, Burdea G, Bouzit M, Popescu V, Deutsch JE. Orthopedic rehabilitation using the "Rutgers ankle" interface. Stud Health Technol Inform [Internet]. 2000 [cited 2016 Jul 18];70:89–95. Available from: http://www.ncbi.nlm.nih.gov/pubmed/10977590
